# Supplementary figures and images for: Comparative Transcriptome and Widely Targeted Metabolome Analysis Reveals the Molecular Mechanism of Powdery Mildew Resistance in Tomato
Source: Int J Mol Sci. 2023 May 4;24(9):8236. doi: 10.3390/ijms24098236 (PMC10178879; doi:10.3390/ijms24098236)

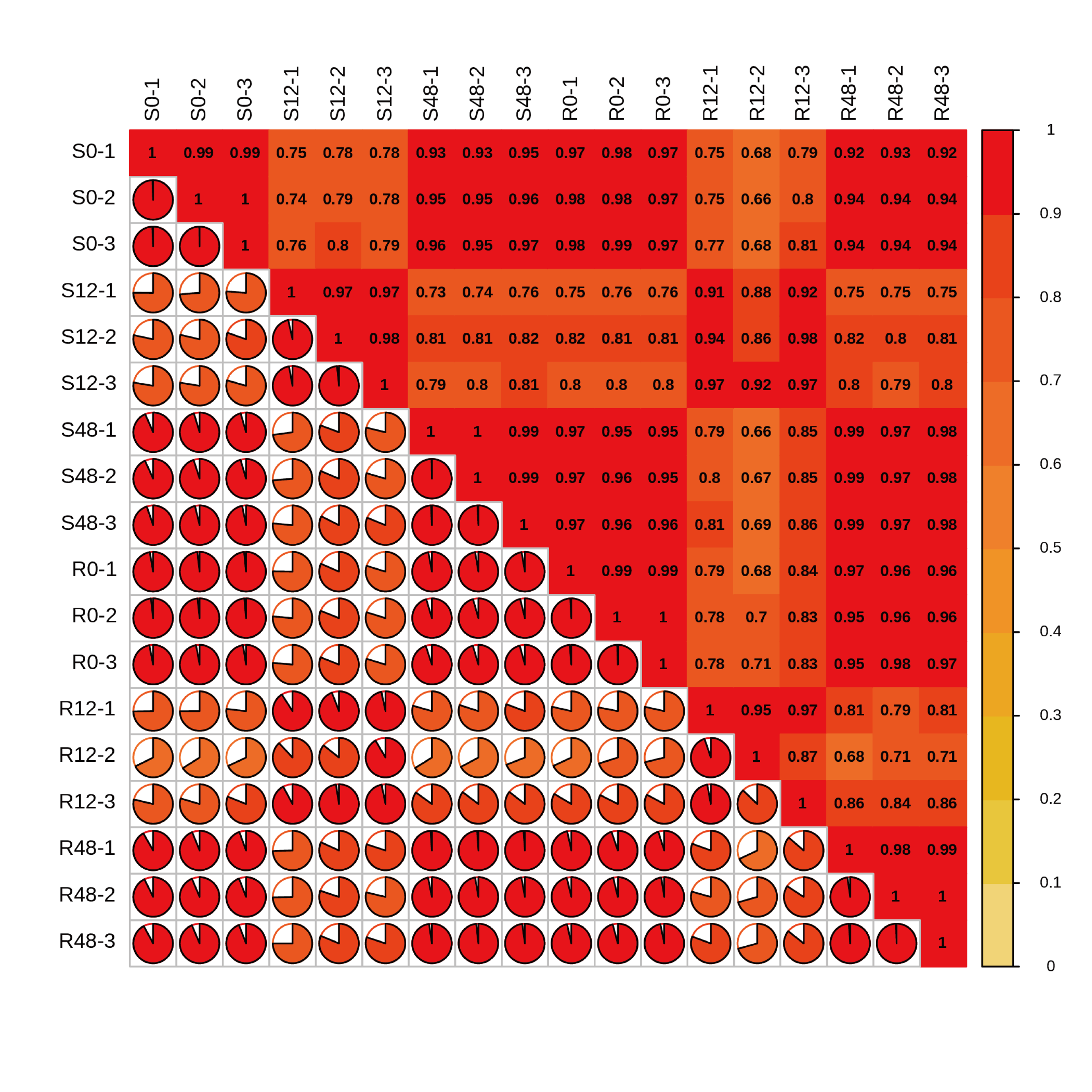

Supplement: Supplementary file 1 [file ijms-24-08236-s001.zip › Figure S1.tif]

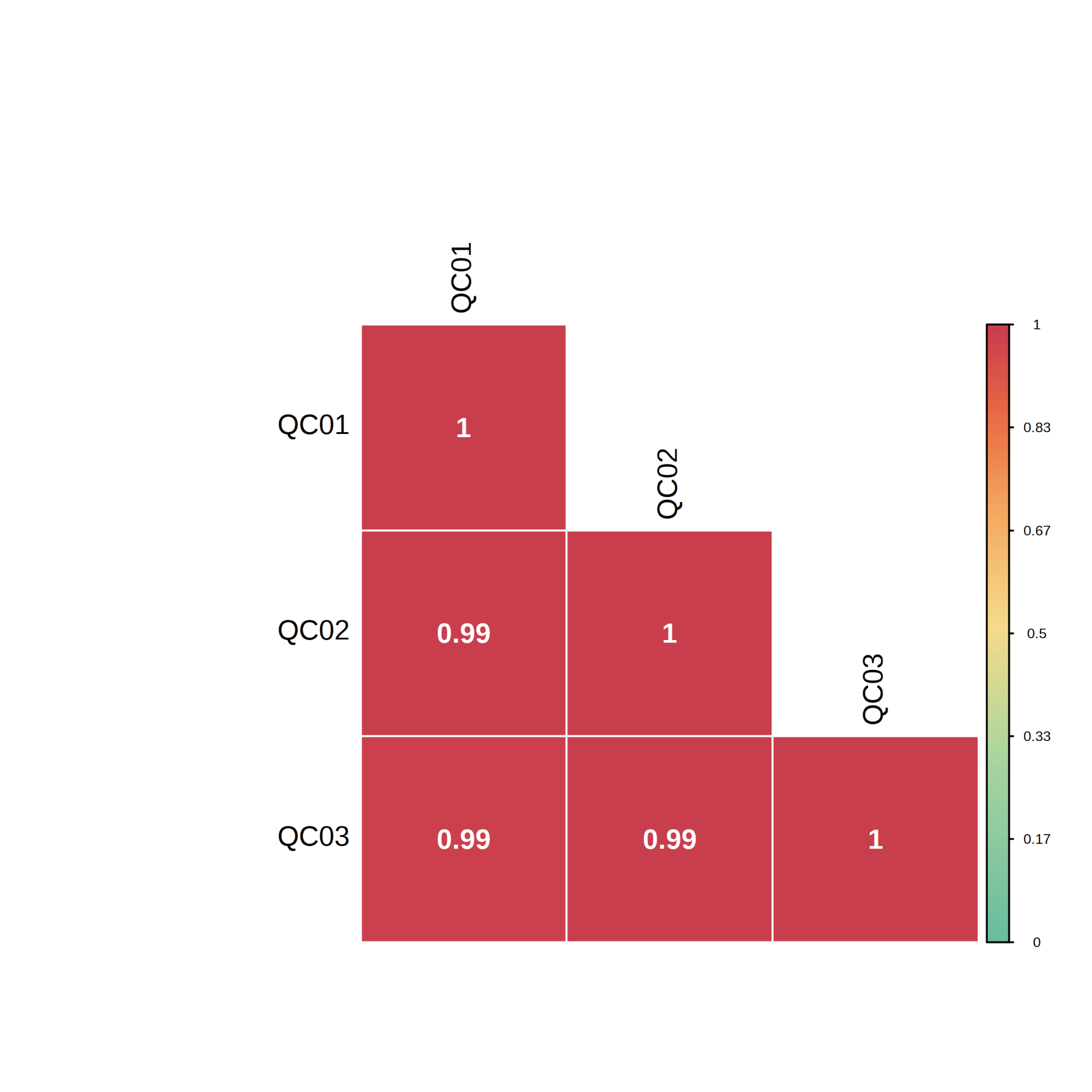

Supplement: Supplementary file 1 [file ijms-24-08236-s001.zip › Figure S10.tif]

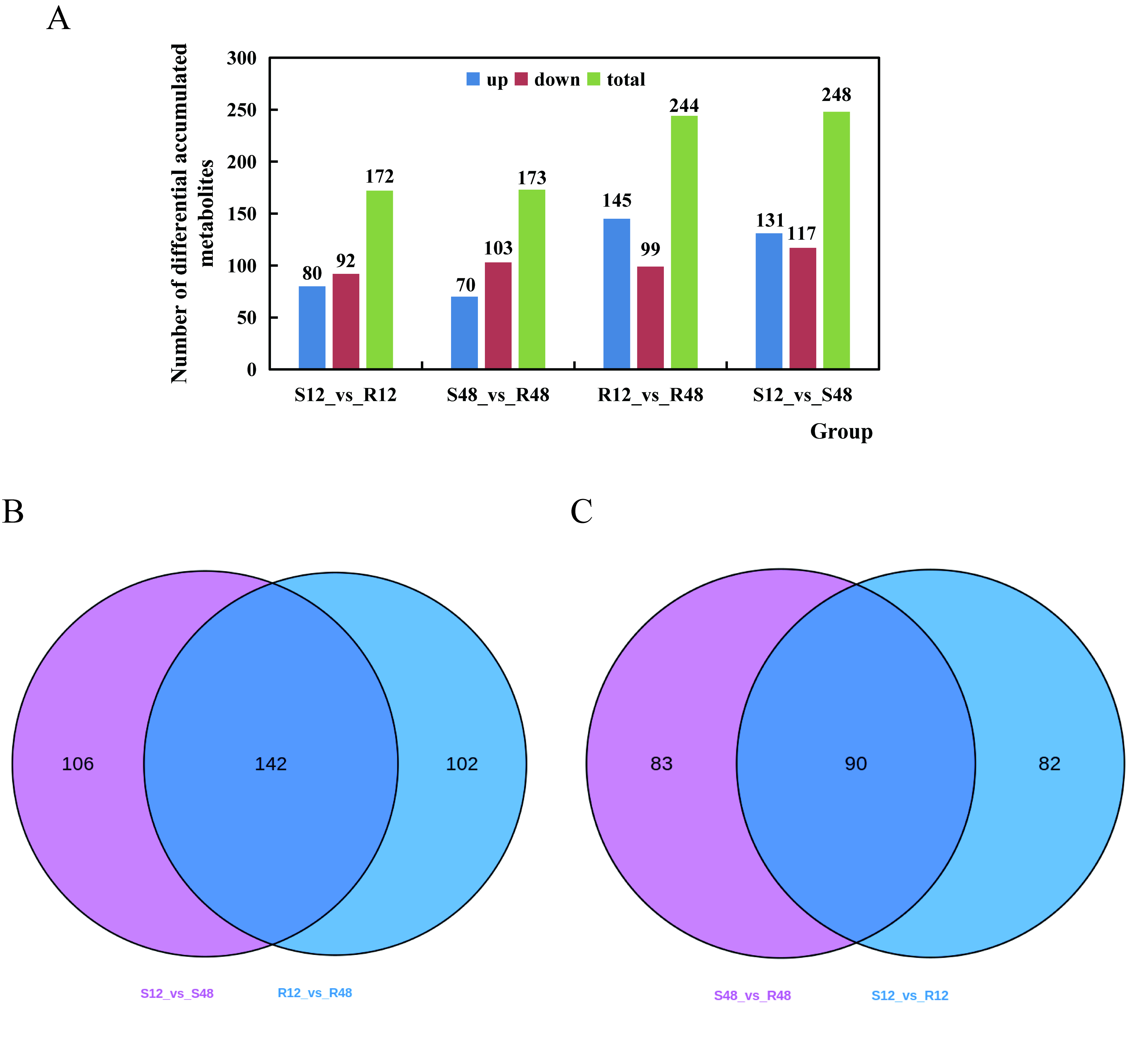

Supplement: Supplementary file 1 [file ijms-24-08236-s001.zip › Figure S11.tif]

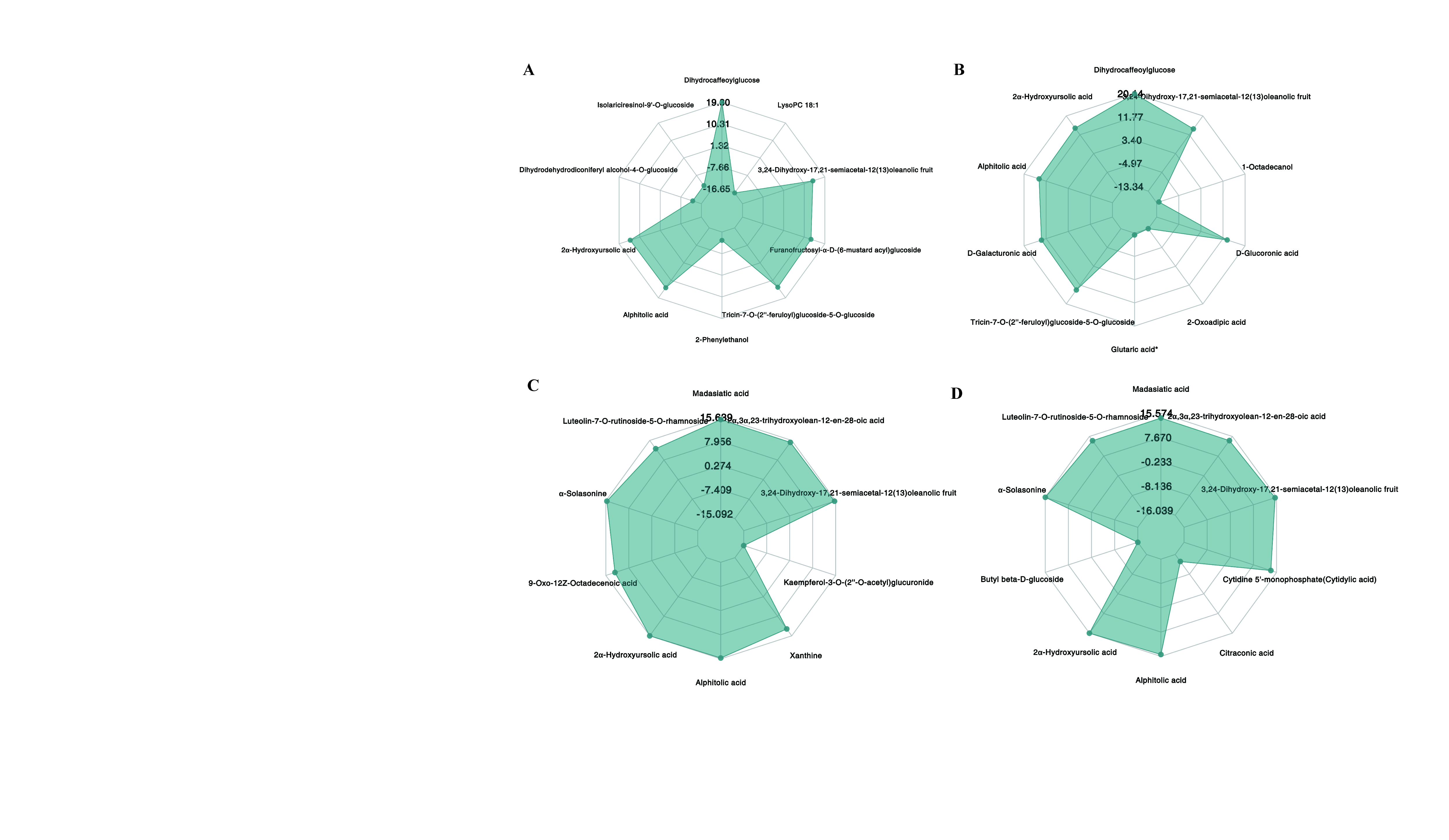

Supplement: Supplementary file 1 [file ijms-24-08236-s001.zip › Figure S12.tif]

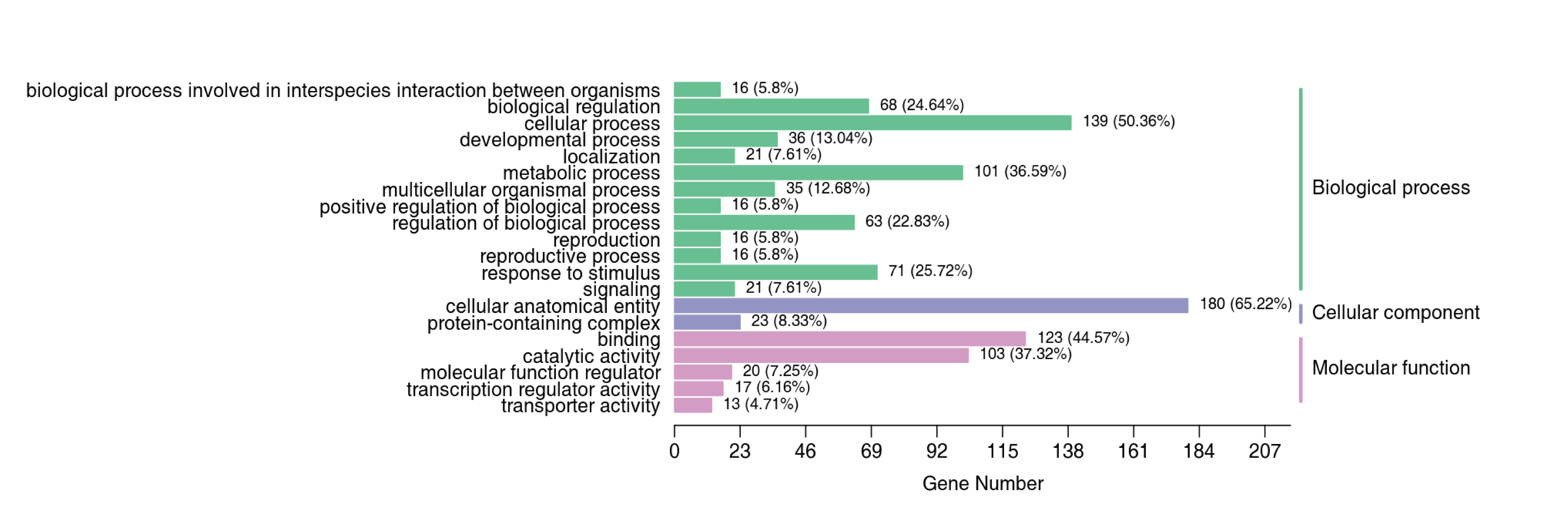

Supplement: Supplementary file 1 [file ijms-24-08236-s001.zip › Figure S3.tif]

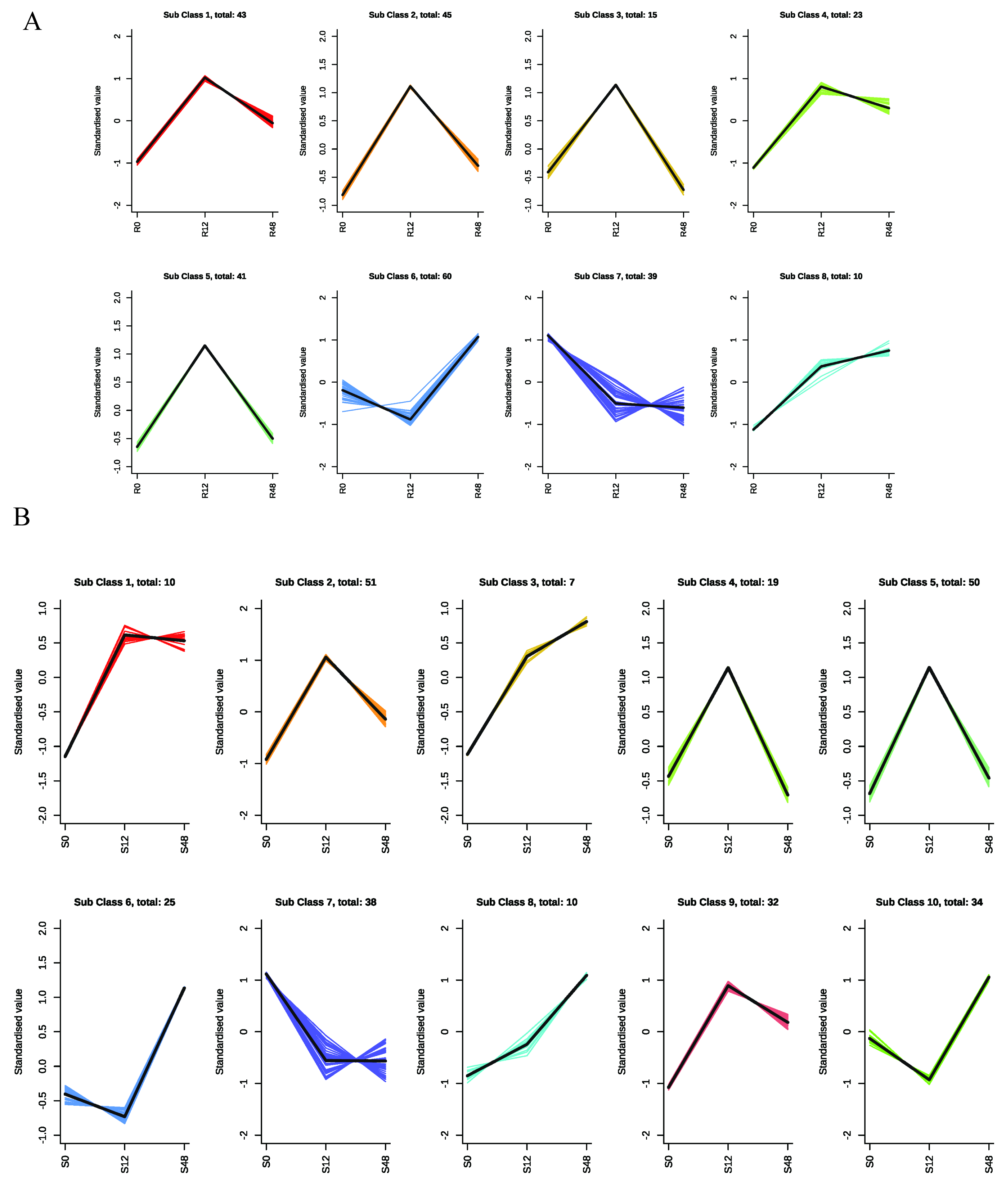

Supplement: Supplementary file 1 [file ijms-24-08236-s001.zip › Figure S4.tif]

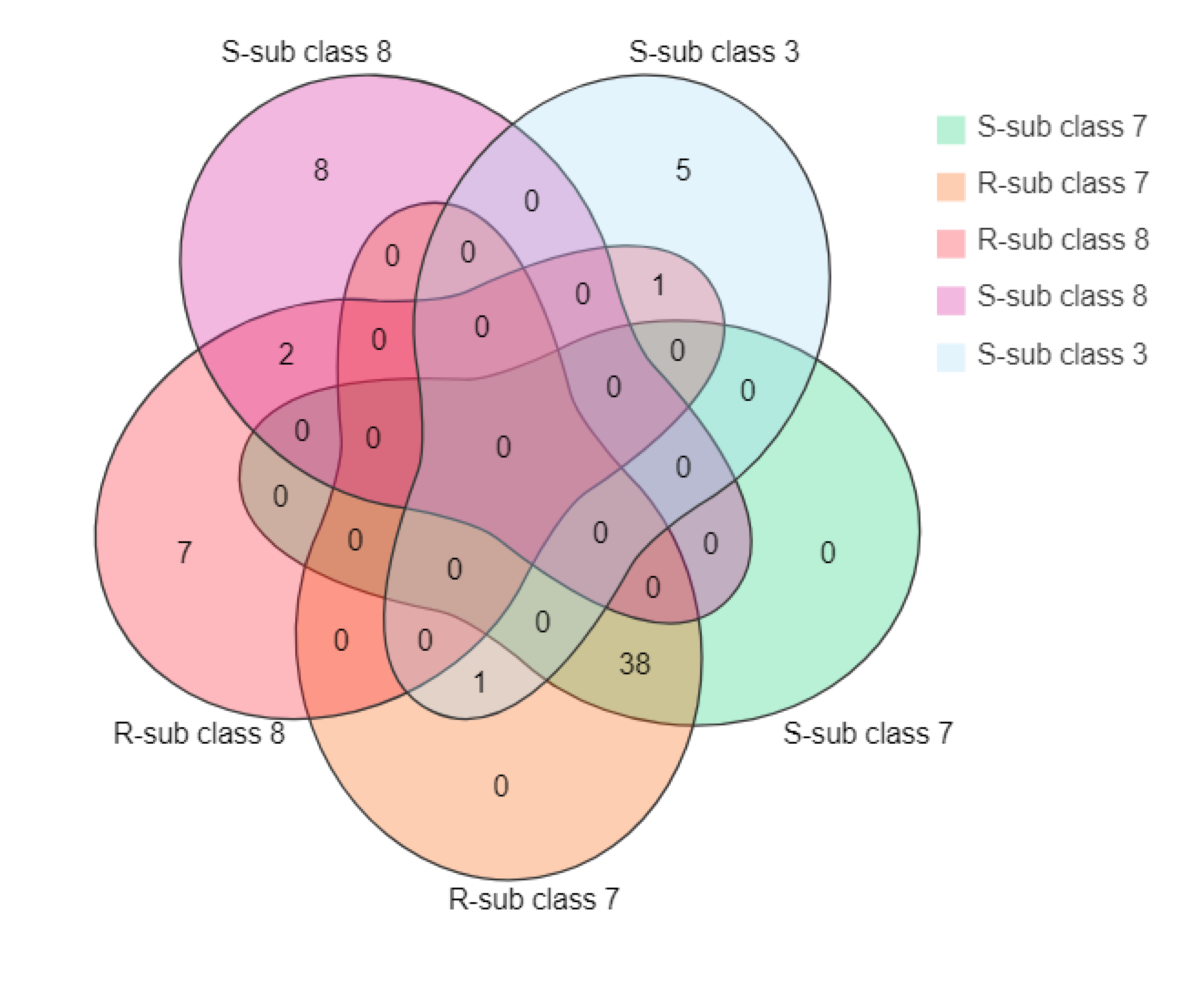

Supplement: Supplementary file 1 [file ijms-24-08236-s001.zip › Figure S5.tif]

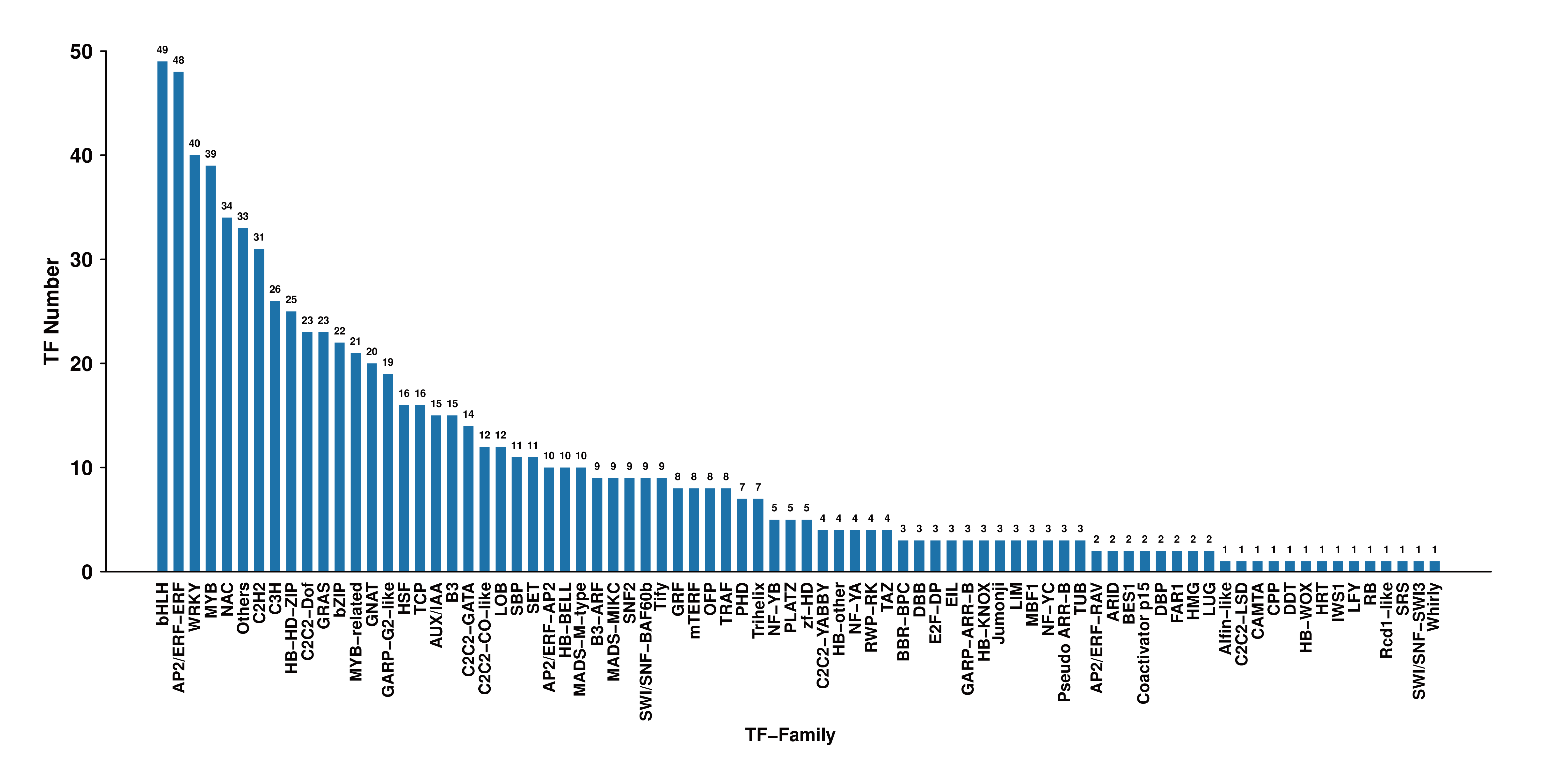

Supplement: Supplementary file 1 [file ijms-24-08236-s001.zip › Figure S6.tif]

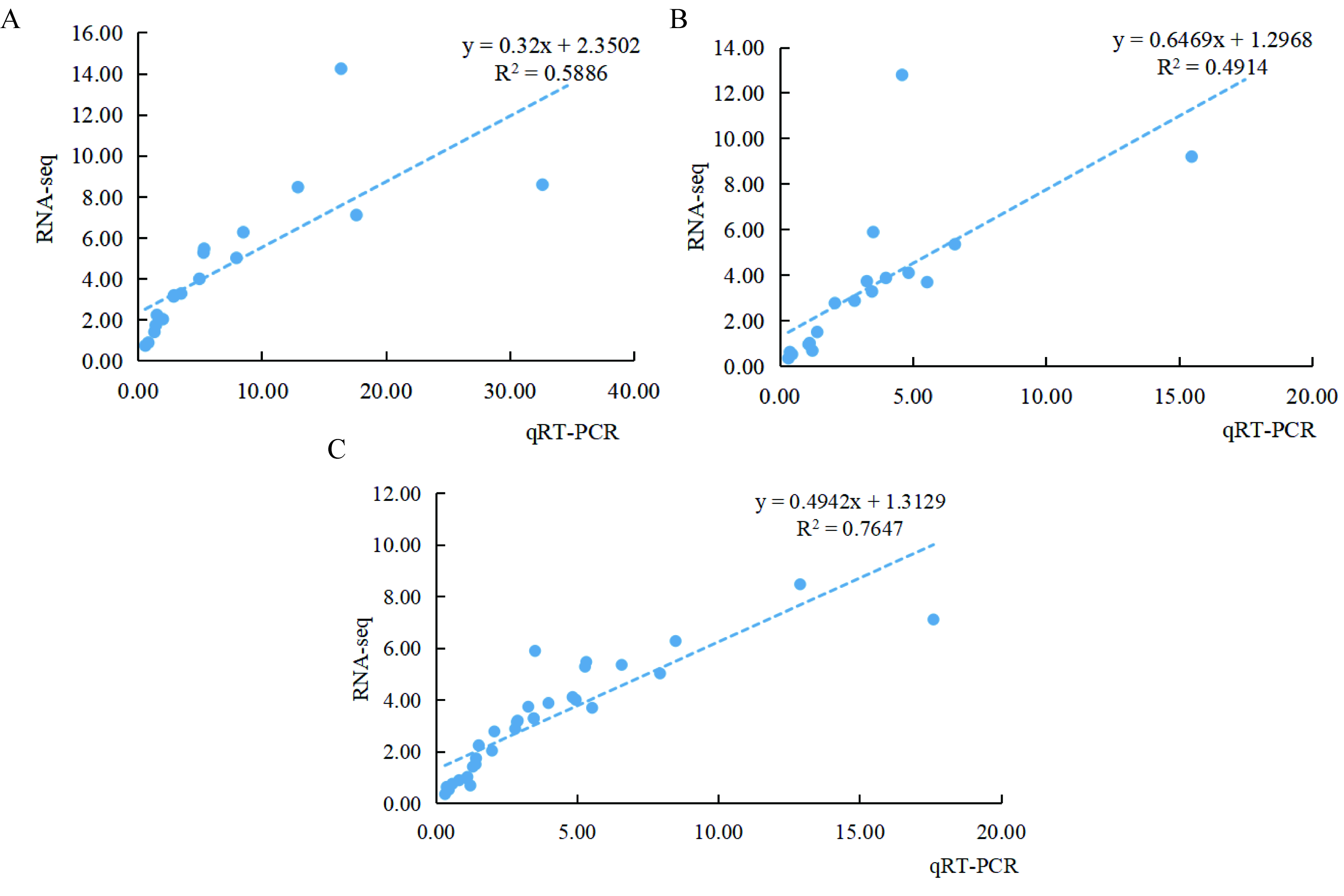

Supplement: Supplementary file 1 [file ijms-24-08236-s001.zip › Figure S7.tif]

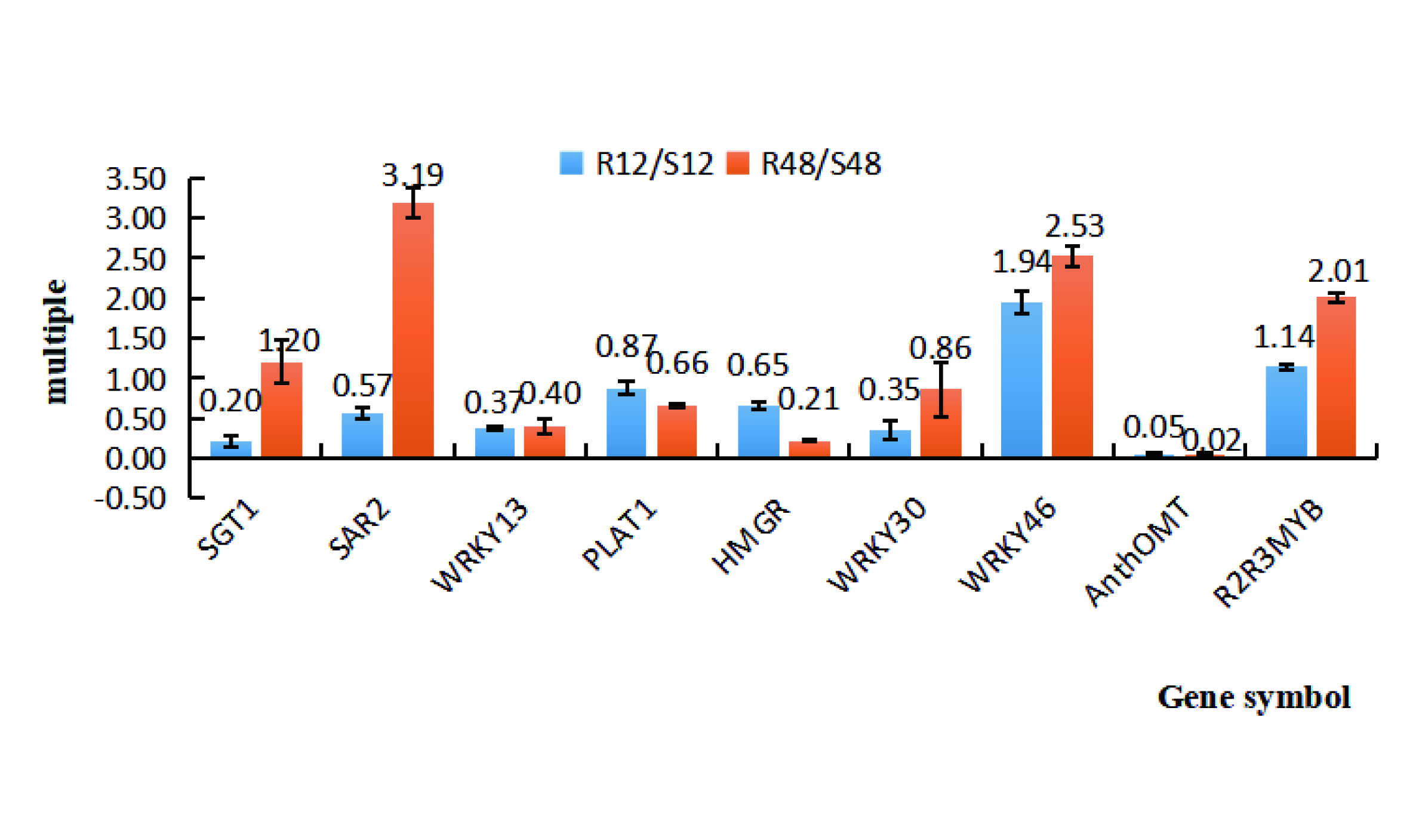

Supplement: Supplementary file 1 [file ijms-24-08236-s001.zip › Figure S8.tif]
